# Stiffness and thickness of the upper trapezius muscle increase after repeated climbing bouts in male climbers

**DOI:** 10.7717/peerj.14409

**Published:** 2022-12-06

**Authors:** Sebastian Klich, Adam Kawczyński, Klaudia Sommer, Natalia Danek, César Fernández-de-las-Peñas, Lori A. Michener, Pascal Madeleine

**Affiliations:** 1 Department of Paralympic Sport, Wrocław University of Health and Sport Sciences, Wrocław, Poland; 2 Wroclaw University of Health and Sport Sciences, Wrocław, Poland; 3 Department of Physiology and Biochemistry, Wrocław University of Health and Sport Sciences, Wrocław, Poland; 4 Department of Physical Therapy, Occupational Therapy, Rehabilitation and Physical Medicine, Universidad Rey Juan Carlos, Madrid, Spain; 5 Cátedra Institucional en Docencia, Clínica e Investigación en Fisioterapia: Terapia Manual, Punción Seca y Ejercicio Terapéutico, Universidad Rey Juan Carlos, Madrid, Spain; 6 Division of Biokinesiology and Physical Therapy, University of Southern California, Los Angeles, United States of America; 7 Department of Health Science and Technology, Sport Sciences—Performance and Technology, Aalborg University, Aalborg, Denmark

**Keywords:** Stiffness, Thickness, Fatigue, Overhead, Shoulder

## Abstract

**Background:**

Indoor climbing involves overloading the shoulder girdle, including the rotator cuff and upper trapezius muscles. This on the field study aimed to investigate the effects of repeated climbing bouts on morphological and mechanical measures of the upper trapezius muscle.

**Materials and Methods:**

Fifteen experienced male climbers participated in the study. Rate of perceived exertion (RPE), blood lactate concentration ([La^−^]_b_), and stiffness and thickness over four points of the upper trapezius were assessed before and after a repeated climbing exercise. The procedure for the climbing exercise consisted of five climbs for a total time of 5-minutes per climb, followed by a 5-minute rest.

**Results:**

The analysis showed an increase from baseline to after the 3rd climb (*p* ≤ 0.01) for RPE and after the 5th climb for [La^−^]_b_ (*p* ≤ 0.001). Muscle stiffness and thickness increased at all points (1–2–3–4) after the 5th climb (*p* ≤ 0.01). We found spatial heterogeneity in muscle stiffness and thickness; muscle stiffness was the highest at Point 4 (*p* ≤ 0.01), while muscle thickness reached the highest values at points 1–2 (both *p* ≤ 0.01). Moreover, the analysis between the dominant and non-dominant shoulder showed greater stiffness after the 1^st^ climb at Point 1 (*p* = 0.004) and after the 5^th^ climb at Point 4 (*p* ≤ 0.001).

**Conclusions:**

For muscle thickness, the analysis showed significant changes in time and location between the dominant and the non-dominant shoulder. Bilateral increases in upper trapezius muscle stiffness and thickness, with simultaneous increases in RPE and blood lactate in response to consecutive climbs eliciting fatigue.

## Introduction

Indoor climbing is gaining popularity among people of different ages and levels of physical activity. Climbing is a versatile sport that engages different motor abilities, *e.g.*, speed, agility, coordination, and balance (Kim & Kim, 2018). Climbing also requires physical performance involving muscle strength, endurance, and flexibility (Grant et al., 2001; Grant et al., 1996; Mermier et al., 2000). However, only a few studies have reported greater flexibility of the upper extremity and endurance of the shoulder girdle musculature in climbers compared with non-climbers (Grant et al., 2001; Grant et al., 1996). Such muscular adaptations augment the risk of muscular overload, due to overhead limb activity (Zago et al., 2020), and potential injury of the trapezius muscle (Roseborrough & Lebec, 2007).

The analysis of the literature shows that only a few studies have contributed to understanding the physiological processes behind indoor climbing (Draper et al., 2011; Gáspari et al., 2015). From a physiological point, metabolic changes measured by blood lactate accumulation might be crucial in the development of specific training performance (Gáspari et al., 2015). Findings from previous studies have demonstrated an increase in lactate concentration immediately after indoor and outdoor climbing as a sign of fatigue development climbing (Draper et al., 2011; Gáspari et al., 2015). It should be noted that changes in lactate concentration have not been previously investigated in relation to morphological properties and stiffness of skeletal muscles.

The functional implications of the trapezius muscle make it the most important muscle of the shoulder region (Madeleine, 2010). The trapezius muscle plays both a postural and a functional role, *i.e.,* it stabilizes the shoulder girdle and contributes to the elevation of the shoulder and rotation of the glenoid fossa as well as adduction of the scapula (Inman, Saunders & Abbott, 1944). Alterations in upper trapezius muscle stiffness and thickness have been reported after upper trapezius muscle eccentric exercises (Kawczynski et al., 2018; Kisilewicz et al., 2020), and repeated shoulder external and internal shoulder rotation exercises (Klich et al., 2020). Muscle stiffness has been evaluated using a hand-held myotonometer (Kawczynski et al., 2018; Kisilewicz et al., 2020; Klich et al., 2020) and shear wave elastography (Kisilewicz et al., 2020). Those studies have reported an acute increase in stiffness of the trapezius muscle in a single point on the muscle belly (Klich et al., 2020) and over four muscle locations (Kisilewicz et al., 2020) measured immediately after eccentric exercise and repeated external and internal rotation. On the contrary, Kawczynski et al. (2018) observed both decreased stiffness over muscle belly locations and increased stiffness over the side of the musculotendinous location after eccentric exercise. All in all, the current findings call for further studies clarifying the effects of fatigue on trapezius muscle stiffness.

Muscle stiffness and thickness alterations expressing morphological changes might increase our understanding of mechanisms behind the development of fatigue of the climber’s upper trapezius muscle. A repeated exercise protocol, performed *in situ* can be used as fatigue testing for the neck and shoulder (Emery & Côté, 2012). Therefore, the aim of this on the field study was to investigate the effects of repeated climbing bouts on morphological and mechanical measures of the upper trapezius muscle. Specifically, we hypothesized an increase in stiffness and thickness of the upper trapezius muscle because of an increase in fatigue-related indicators (rate of perceived exertion and blood lactate concentration), after repeated climbing exercise.

## Materials & Methods

### Participants

This observational, case series study assessed morphological, mechanical, and fatigue-related measures after five repeated trials of a 15-meter indoor climbing exercise. The eligible participants (*n* = 15) consisted of male recreational indoor climbers. All description about participant demographics, training experience, and duration is included in Table 1. The entire group was right-hand dominant with their best climbing grade at level 6a+ (achieved in the past 2 years). The level of the route was based on the climber’s skills and performance, according to the French climbing grade scale (Draper et al., 2011). The inclusion criteria were: (1) training experience in indoor climbing ≤5 years, (2) training duration ≥8 h per week, and (3) the best level in climbing at 6a+. Exclusion criteria were: (1) strength training 2 weeks prior to the experiment, (2) trauma or surgery, and (3) shoulder pain in the past 6 months.

A power analysis was conducted using G*Power software (version 3.1.9.2; Kiel University, Kiel, Germany) (Faul et al., 2007) for a repeated measure of analysis of variance (RM-ANOVA) within factors. For upper trapezius stiffness and thickness measures, a minimum expected “medium” effect size (Cohen’s *f*) of 0.35, an *α* level of 0.05, a power of 0.90, and a correlation for repeated measures of 0.6 (for stiffness and thickness measurements). The procedure included a minimum number of 11 participants, but a total of 15 were recruited to account for potential dropouts.

All participants read and signed an informed consent form approved by the Senate Research Ethics Committee at the Wrocław University of Health and Sport Sciences (project identification code: 1/2019 approval date: 11.01.2019). The study was conducted according to the Declaration of Helsinki.

### Experimental procedures

**Figure 1:**
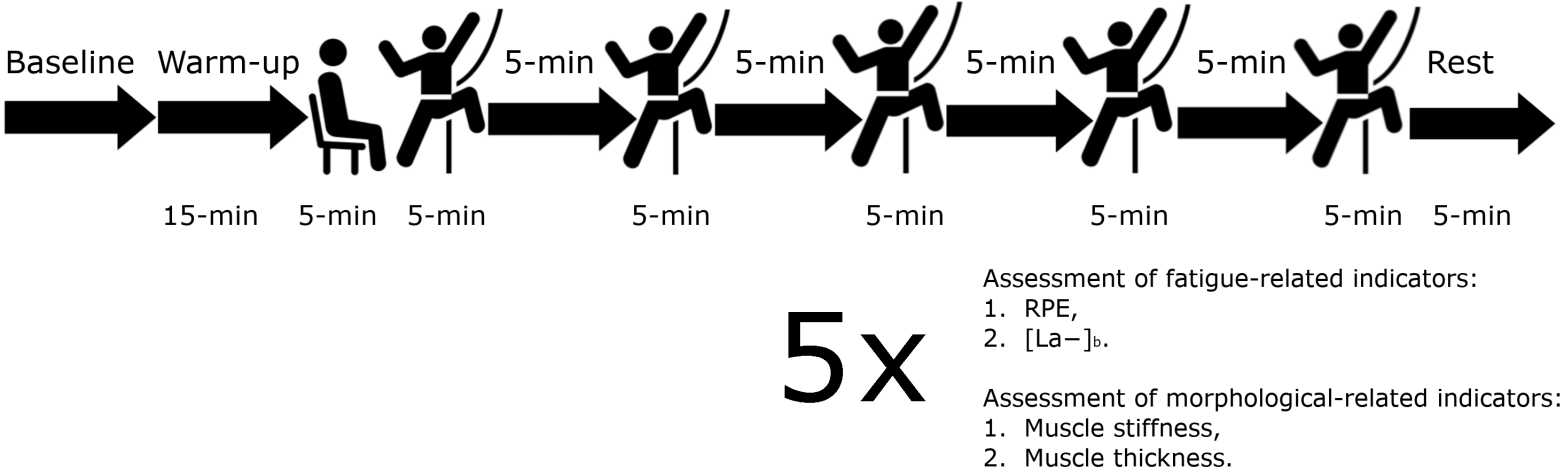
The experimental procedure includes measurements taken at baseline and cool dawn including rate of perceived exertion (RPE), blood lactate concentration ([La-]), myotonometry and ultrasonography. After each 5-min climb measurement of RPE, myotonometry and ultrasonography were taken.

Participants underwent baseline measures of the upper trapezius, *i.e.,* stiffness and thickness, rate of perceived exertion (RPE), and blood lactate level. Data were collected as previously described by Klich et al. (2022). Specifically, all potential participants were informed to refrain from strength training, including climbing within 48 h prior to the experiment. The climbing tasking first involved a 15-minute warm-up, followed by a testing climb at 6a+ and a passive rest (5-minutes). The main goal of this climbing tasking was to provoke acute fatigue-induced alterations in the upper trapezius muscle. The procedure for the climbing exercise was based on five climbs (5-minutes for each) and a 5-minute rest after each bout (Fig. 1).

**Table 1:** Mean ± SD of the participant demographics, training experience, and duration.

Variables	Recreational indoor climbers *n* = 15
Age (year)	28.8 ± 7.4
Body height (cm)	178.4 ± 9.3
Body mass (kg)	74.5 ± 6.7
Body Mass Index (kg/m^2^)	23.7 ± 1.5
Training experience (year)	6 ± 2
Training duration (hours/week)	10 ± 1

Mechanical and morphological measurements consisted of upper trapezius stiffness and thickness, and fatigue-related measurement (RPE) at baseline and immediately after each of the five climbs. Fingertip blood lactate concentration ([La^−^]_b_) was measured at baseline and after the fifth climbing exercise in the third minute of the rest period. All measurements were performed during the passive rest (5 min); however, not later than 30 s after the end of each climbing bout. Data were collected in the following order: (1) RPE, (2) stiffness of the upper trapezius muscle, (3) thickness of the upper trapezius muscle, and (4) [La^−^]_b_. Muscle morphological measurements were taken on both shoulders six times; at baseline and immediately after five climbs.

### Rate of perceived exertion

The Borg’s Rate of Perceived Effort (RPE) scale was used to measure the level of exertion (Borg, 1998). The RPE is a tool for the subjective assessment of exercise intensity during activity, *i.e.,* how hard they feel they are working. The RPE scale ranges from 6 to 20, with 6 indicating no exertion and 20 indicating maximal exertion. In general, a score >18 indicates that a maximal effort was made, and values >15–16 indicate that the anaerobic threshold was exceeded.

### Blood lactate concentration

Blood was collected from the climber at rest prior to starting the protocol, and at the third minute of rest, after the 5th climb. The samples were drawn from the fingertip of both hands, using a disposable Medlance^^®^^ Red lancet spike (HTL-Zone, Berlin, Germany). The procedure was performed to determine blood lactate concentration ([La^−^]_b_) (mmol l^−1^) with a Lactate Scout 4 analyzer (EKF Diagnostics, Magdeburg, Germany) at baseline and the end of the climbing protocol.

### Upper trapezius stiffness and morphology

**Figure 2:**
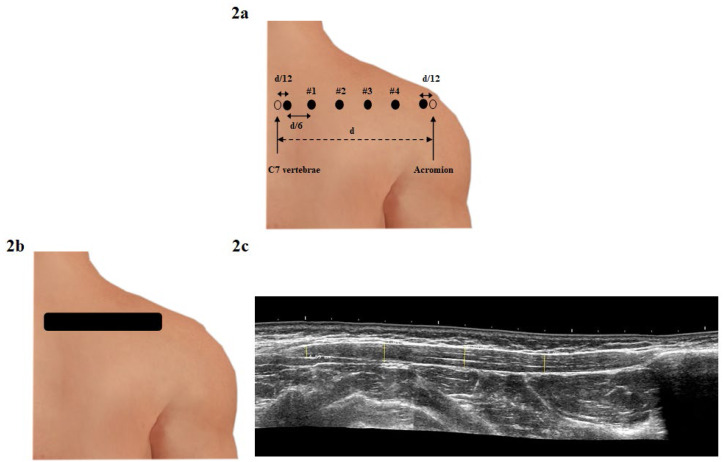
Evaluation of stiffness and thickness of the upper trapezius muscle: (A) Reference points and locations including four points for measuring the upper trapezius muscle stiffness according to Kisilewicz et al. (2020). In this figure “d” indicates the distance between the spinous process of the C7 vertebra and acromion, while “d/12” is “d” divided by 12; “d/6” is “d” divided by 6; (B) ultrasound measurement of the upper trapezius muscle thickness on the long axis, including the measure in four reference points; (C) ultrasound transducer position to the muscle fibers located of the C7 vertebra.

The stiffness of the upper trapezius muscle was measured with a hand-held myotonometer (MyotonPro, Myoton Ltd, Tallinn, Estonia). Participants were seated on a comfortable chair with the lower back supported by the chair back and forearms supported on the desk. A wax pencil was used to mark the four locations for the muscle stiffness measures. First, the distance between the spinous process of the C7 vertebra and acromion was measured (mean 24.4 ± 1.2 cm) and then divided by 6 (mean 4.1 ± 0.5 cm) to calculate the marker placement. The most lateral points from the C7 vertebra were divided by 12 (mean 2.0 ± 0.3 cm) and were not included in data collection because those points correspond to the musculotendinous site of the upper trapezius (Kawczynski et al., 2018). The four medial points (#1 –4) were used for measurement because were considered muscle belly sites (Fig. 2A) (Kawczynski et al., 2018; Kisilewicz et al., 2020). Next, the myotonometer probe was placed perpendicular to the designated four measurement points, and then the probe generated three impulses (0.4 N for 15 ms) exerted on the area (Kelly et al., 2018). Reliability was excellent for stiffness analyzed for Point 2 (ICC_2,1_ = 0.92); SEMs was 26 N/m, while MDC90% was 39 N/m.

Muscle thickness of the upper trapezius was measured using ultrasonography (Alpinion X-CUBE 90; Alpinion, Seoul, South Korea) with a 10.0 (3.0 to 19.0) MHz and 60 mm linear array transducer (Single Crystal; Alpinion, Seoul, South Korea) in grey-scale B-mode. A single examiner obtained ultrasound images of the trapezius muscle on a long axis using the longitudinal view feature to obtain an image of the full length of the upper trapezius muscle. The participants were seated on a comfortable chair with their lower back supported on the chair back and forearms supported on the desk. The linear transducer was positioned longitudinal to the muscle fibers located in the C7 vertebra and moved distally to the acromion in a strength line (Fig. 2B) (Kisilewicz et al., 2020). Measurements of upper trapezius thickness were performed using MicroDicom viewer software (MicroDicom DICOM Viewer, Bulgaria). To obtain thickness for four reference points, four parallel lines were located between the superficial and deep fascia of the upper trapezius muscle belly (Kisilewicz et al., 2020). Each measurement was taken twice and averaged for data analysis (Fig. 2C). The intra-examiner reliability was good for a single reference point (ICC_2,1_ = 0.89); SEMs were 0.4 mm and the MDC90% was 0.4 mm.

### Statistical analysis

The SPSS 18 statistical software (SPSS Inc., Chicago, Illinois, USA) was used for data analysis. Mean values ± standard deviation (SD) as well as mean differences with confidence intervals (CI 95%) were calculated. The normality of the data distribution was assessed with the Shapiro–Wilk tests, while homogeneity of variance was analyzed by Levene’s test. The analyzed data were normally distributed for all parameters, while the variances for all parameters were equal. A one-way analysis of variance with repeated measure (RM-ANOVA) with t*ime* (baseline–climb 1–5) was used as a within-subject factor for differences in RPE and [La^−^]_b_. Moreover, a three-way RM-ANOVA with *time* (baseline–climb 1–5), *side* (non-dominant –dominant), and *location* (points 1–2–3–4) was conducted for upper trapezius stiffness and thickness. If a significant interaction between variables was found, the Bonferroni adjustment for multiple comparisons was used for post hoc tests (*p* ≤ 0.01). The effect size was estimated using partial eta square (*η*^2^), classified as small (0.2<*η*^2^<0.49), medium (0.5<*η*^2^<0.79), or large (*η*^2^ ≤0.8) (Richardson, 2011). For all statistical tests, a *p*-value <0.05 was considered significant.

## Results

### Fatigue-related indicators

**Figure 3:**
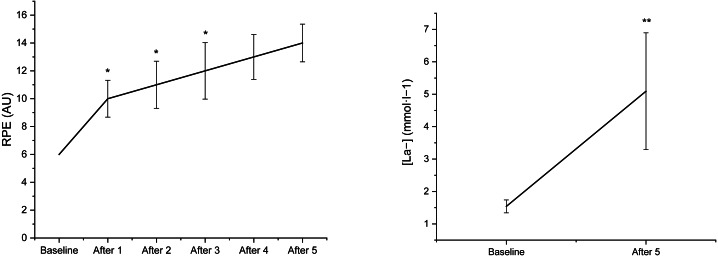
Mean ± SD of the rate of perceived exertion (RPE) and blood lactate concentration [La^−^] over time in indoor climbers after an exercise protocol composed of five climbs. An asterisk (*) indicates significant changes compared with prior climb (Climb 1 *vs* Baseline; Climb 2 *vs* Climb 1; Climb 3 *vs* Climb 2; Climb 4 *vs* Climb 3 and Climb 5 *vs* Climb 4) (*p* < 0.01) for RPE. ** Significant differences compared with baseline (*p* < 0.01) for [La^−^].

Figure 3 reports the mean   ±  SD of the RPE at baseline and after each climbing, exercise as well as [La^−^]_b_ at baseline and after the 5th climb. The one-way RM-ANOVA revealed an effect of *Time* in RPE (*F*_5,70_ = 126.2, *p* ≤ 0.001, *η*^2^ =0.90). The post-hoc analysis showed an increase from baseline to 5th climbing exercise for RPE (*p* < 0.05 for all). The one-way RM-ANOVA revealed an effect of *Time* for [La^−^]_b_ (*F*_1,14_ = 23.5, *p* ≤ 0.001, *η*^2^ = 0.63). The post-hoc analysis showed a gradual increase for RPE (*p* ≤ 0.01) for RPE and after the 5th climb for [La^−^]_b_ (*p* = 0.001) (Fig. 3).

### Trapezius muscle stiffness

**Figure 4:**
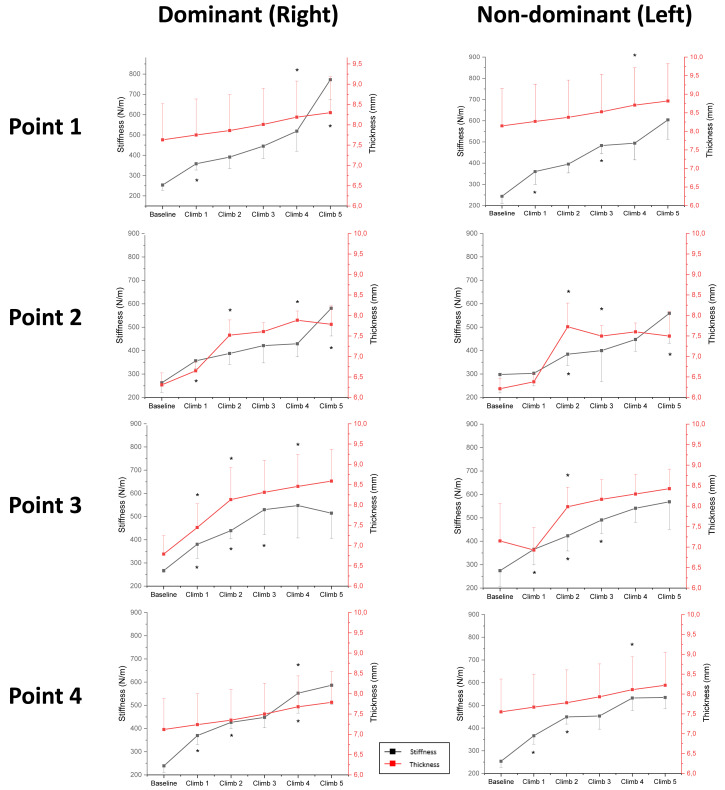
Mean ± SD of the stiffness (N/m) and thickness. Dominant (Right) Non-dominant (mm) of the dominant and non-domin. An asterisk (*) indicates significant changes compared with prior climb (Climb 1 *vs* Baseline; Climb 2 *vs* Climb 1; Climb 3 *vs* Climb 2; Climb 4 *vs* Climb 3 and Climb 5 *vs* Climb 4) (*p* < 0.01).

**Figure 5:**
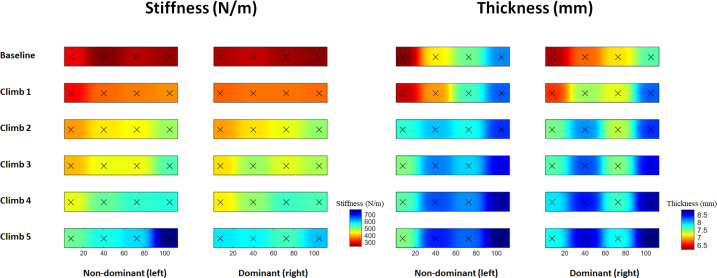
Topographical maps of the mean muscle stiffness (N/m) and thickness (mm) of the dominant and non-dominant upper trapezius muscle over time in indoor climbers after an exercise protocol composed of five climbs.

Figures 4 and 5 shows the mean   ±  SD of the upper trapezius muscle stiffness and thickness at baseline and after each repeated climbing exercise. The three-way RM-ANOVA revealed a main effect of *Time* (*F*_5,550_ = 346.4, *p* ≤ 0.001, *η*^2^ =.076) and *Location* (*F*_3,112_ = 10.2, *p* ≤ 0.001, *η*^2^ = 0.22) for upper trapezius stiffness Moreover, the analysis revealed an interaction effect between *Side* and *Time* (*F*_5,550_ = 2.3, *p* =0.034, *η*^2^ = 0.22), *Time* and *Location* (*F1*_5,550_ = 9.8, *p* ≤ 0.001, *η*^2^ = 0.21), as well as *Side* and *Location* and *Time* (*F1*_5,550_ = 3.4, *p* ≤ 0.001, *η*^2^ =0.08) for upper trapezius stiffness. Muscle stiffness increased at all points (1–2–3–4) after 1st climb (*p* ≤ 0.001 for all), as well as at Point 2 and Point 3 after 2nd climb (*p* ≤ 0.001 for both) and 3rd climb (*p* ≤ 0.001 at Point 2) in the dominant shoulder. There were increases in muscle stiffness at Point 3 after the 4th climb (*p* = 0.003) and at Point 4 after the 5th climb (*p* ≤ 0.001) in the dominant shoulder. Moreover, increases in muscle stiffness were observed at Point 2 after the 3rd climb and 4th climb, as well as at Point 3 after the 4th climb, compared with Point 1 after the 3rd climb (*p* = 0.001) and 4th climb (*p* = 0.001) in the dominant shoulder. Increases in muscle stiffness were also seen at Point 4 after the 5th climb compared with Point 1–2–3 after the 5th climb (*p* ≤ 0.001 for all) in the dominant shoulder. Additionally, muscle stiffness increased at Points 2–3–4 after 1st climb (*p* ≤ 0.001 for all), as well as at Points 1–2–3 after 2nd climb (*p* ≤ 0.001 for all) in the non-dominant shoulder. There were increases in muscle stiffness at Point 2 and Point 4 after the 3rd climb (*p* = 0.01 and *p* = 0.001, respectively), and at Point 1 after the 5th climb (*p* = 0.01) in the non-dominant shoulder. Moreover, decreases in muscle stiffness were observed at Point 1 after 1st climb compared with Point 2–3–4 after 1st climb (*p* = 0.01 for all) in the non-dominant shoulder. Increased muscle stiffness was found at Point 3 after the 2nd climb, and at Point 2 after the 3rd climb, compared with Point 1 after the 2nd climb (*p* = 0.001) and 3rd climb (*p* = 0.01) in the non-dominant shoulder (Fig. 4). Finally, the analysis showed greater stiffness after the 1st climb at Point 1 (*p* = 0.004) and after the 5th climb at Point 4 (*p* ≤ 0.001) in the dominant, compared with the non-dominant shoulder (Figs. 4 and 5).

### Trapezius muscle thickness

The three-way RM-ANOVA revealed a main effect of *Time* (*F*_5,560_ = 271.3, *p* ≤ 0.001, *η*^2^ =0.71) and *Location* (*F*_3,112_ = 11.7, *p* ≤ 0.001, *η*^2^ = 0.24) for upper trapezius thickness. Moreover, the analysis revealed an interaction effect between *Time* and *Side* (*F*_5,550_ = 3.1, *p* = 0.028, *η*^2^ = 0.27), *Time* and *Location* (*F1*_5,550_ = 23.2, *p* ≤ 0.001, *η*^2^ =0.38), as well as *Side* and *Location* and *Time* (*F1*_5,550_ = 2.3, *p* ≤ 0.02, *η*^2^ = 0.08) for upper trapezius thickness. We found an increase in muscle thickness at Point 2 after 1st climb (*p* ≤ 0.001), Point 1–2 after 2nd climb (*p* ≤ 0.001 for both), as well as Point 1–2–3–4 after 4th climb (*p* ≤ 0.01 for all) in the dominant shoulder. Moreover, decreases in muscle thickness were observed at Point 1 after the 2nd climb and 4th climb, compared with Point 2 (*p* = 0.01) and Point 4 (*p* ≤ 0.001) after the 2nd climb in the dominant shoulder. There were increases in muscle thickness at Point 1 and Point 2 after the 2nd climb (*p* ≤ 0.001 for both), at Point 1 after the 3rd climb (*p* = 0.008), and at Point 3 and Point 4 after the 4th climb (*p* ≤ 0.001 for both) in the non-dominant shoulder. Moreover, decreases in muscle thickness were seen at Point 1 after 1st climb compared with Point 2 and Point 4 after 1st climb (*p* ≤ 0.001 for both) in the non-dominant shoulder. Increased muscle thickness was found at Point 4 after the 3rd–4th–5th climb, compared with Point 1 after the 3rd–4th–5th climb (*p* ≤ 0.001 for all) in the non-dominant shoulder (Fig. 4). Finally, the analysis showed none changes in time and location between the dominant and the non-dominant shoulder (Figs. 4 and 5).

## Discussion

The current field study revealed that fatigue developed over time (increased RPE and blood lactate acid concentration) during dynamic muscle action occurring during repeated climbing resulting in concomitant alterations of muscle morphological structure and mechanical properties of both trapezius muscles in recreational indoor climbers. Specifically, we observed bilateral increases in upper trapezius muscle stiffness and muscle thickness immediately after each of the five bouts of the climbing exercise. The current study showed that measures of muscle stiffness and thickness are useful when assessing acute morphological alterations of the upper trapezius in relation to repeated climbing exercises.

The present study showed a blood lactate concentration of 5.1 ± 2.7 mmol l^−1^ after the last climb, and a significantly higher RPE with each subsequent attempt confirming that our protocol consisted of five repeated climbs interspaced by five minutes resulting in fatigue development. Previous studies have shown similar findings according to blood lactate concentrations ranging from ∼4.0 mmol l^−1^ to 7.0 mmol l^−1^ (Draper et al., 2011; Gáspari et al., 2015). However, it should be underlined that in those studies, different designs and experimental procedures were performed. Draper et al. (2011) and Gáspari et al. (2015) analyzed the on-sight lead climb and lead the real climbing competition, whereas Watts et al. (2000) after rock climbing. Another factor increasing metabolic response and RPE may have been related to unfamiliarity with the route or to the competition itself (Draper et al., 2008). All in all, the current protocol enables the assessment of acute changes due to fatigue development during repeated climbing.

In this study, we investigated morphological alterations in the upper trapezius muscles, expressed by stiffness and thickness immediately after each climbing exercise. Muscle stiffness increased at all points on both sides after each climbing exercise; the highest increase was observed after 1st (from 24 to 35%) and 2nd (from 8 to 18%) climbing at points 1–4 on both sides, and the lowest after 3rd (from 1 to 5%) climbing at Point 3 on both sides. Our results also showed an increase in upper trapezius muscle thickness after 2nd (from 8 to 17%) climbing at Point 1 and Point 2 bilaterally, as well as a decrease after 5th (−1%) climbing. Increases in both muscle stiffness and thickness occurred as a result of repeated climbing provoking fatigue development. Leong, Hug & Fu (2016) reported that alterations in the morphological properties of the supraspinatus tendon may induce greater upper trapezius muscle stiffness in overhead athletes. The current findings are also in line with our previous study reporting an increase in supraspinatus tendon thickness as well as a simultaneous increase in upper trapezius muscle stiffness after repeated shoulder external and internal rotation exercises (Klich et al., 2020). Acute alterations in upper trapezius stiffness may be associated with changes in muscle tissue density (extracellular matrix) causing an increased rate of collagen turnover (Hjorth et al., 2015). This acute mechanism can cause disturbance in fluid circulation and finally edema (Kjaer, 2004). Andonian et al. (2016) found that higher stiffness after fatiguing exercise may be associated with altered extracellular water volume. Furthermore, the increased upper trapezius muscle thickness could be explained by a relapse of glycosaminoglycans leading to direct alterations in tendon stiffness and due to greater vascularity of the muscle (Tsui et al., 2017). Such changes in morphological structure and mechanical properties seem to underline the risk of injury in the shoulder girdle during overhead movements (Hume, Reid & Edwards, 2006). However, the latter studies do not provide information of the spatial dependencies over changes in the trapezius muscle.

In this current study, we found a consistent spatial distribution in muscle stiffness and thickness of the upper trapezius muscles (Fig. 5). Muscle stiffness was the highest at the most distal location (Point 4), while muscle thickness reached the highest values at Point 1 and Point 2. The observed spatial heterogeneity, in both upper trapezius muscle stiffness and thickness, is in line with previous findings (Ishikawa et al., 2017; Kisilewicz et al., 2020). Those changes might be explained by different factors, *e.g.*, muscle architectonical alterations, fiber type (Agur et al., 2003), and fatigue-induced changes (James & Green, 2012). Moreover, from a physiological perspective, the proximal part of the upper trapezius (Point 1 and Point 2) is characterized by a thicker muscle belly, while the distal part (Point 3 and Point 4) may correspond to a more superficial musculotendinous area (Kawczynski et al., 2018; Madeleine et al., 2018). Our results showed a greater increase in stiffness and thickness at proximal and distal points after 1st climb on both shoulders. A greater increase occurred at proximal points after the 2nd climb and 3rd climbs and at distal points after the 4th climb on both shoulders. Indoor climbing requires bilateral use of both upper extremities but there are functional differences among sides. The dominant arm does one-arm hang (used for rest) and is used when holding holds while the non-dominant arm is preferred for overhead and horizontal movements to reach higher holds (Kozin et al., 2020). The observed larger increase in stiffness and thickness of the dominant compared with the non-dominant side mostly reflected such differences in function. Furthermore, this strategy developed by climbers may also be due to the lower fatigability of the dominant compared with the non-dominant side (Madeleine & Farina, 2008). In conclusion, there seem to be specific stiffness and thickness phenotypes of the dominant and non-dominant upper trapezius muscle underlining that the accrued alterations on the dominant side may put the climber at higher risk of overuse injury (Jones, Asghar & Llewellyn, 2008; Rohrbough, Mudge & Schilling, 2000).

These findings presented in this current study may be important for injury prevention of overhead movements and patterns, *i.e.,* shoulder movement and scapulo-humeral rhythm. Overhead athletes, as climbers require a functional and morphological performance to prevent shoulder pain and impingement syndrome. Repetitive climbing exercises defined as sub-maximal and bilateral contractions may affect the properties of shoulder muscle, especially the trapezius. Control of training loads should be considered focused on an appropriate volume and intensity of climbing training. Thus, the monitoring of the training process, based on fatigue-related indicators and muscle properties, should indicate the appropriate number of repetitions and time for a single climbing bout and rest. In summary, the final findings should be applied by strength and conditioning coaches for the control and monitoring of a training unit, as well as physical therapists and athletic trainers to systemize knowledge for injury prevention programs.

The current study has some limitations. Longer or shorter climbing times could produce different results. Furthermore, future research should include climbers of both sexes. We recruited only male subjects because a control group consisting of women could falsify the results due to hormonal changes during the menstrual cycle. Finally, we tested a small group of participants (*n* = 15), limiting the generalizability of the results to climbers that match the characteristics of our sample only. Future studies could also investigate changes in shoulder function and morphological properties in relation to the specific climbing disciplines, *i.e.,* speed, lead, or bouldering.

## Conclusion

The present study showed, for the first time, changes in morphological properties of the upper trapezius muscle and fatigue-related indicators after repeated exercise protocol in recreational indoor climbers. We found significant bilateral increases in upper trapezius muscle stiffness and thickness, with simultaneous increases in RPE and blood lactate in response to consecutive climbs eliciting fatigue. Interestingly, these increases were more marked on the dominant side suggesting a higher risk of injuries on the dominant side.

##  Supplemental Information

10.7717/peerj.14409/supp-1 Supplemental Information 1 Morphological, mechanical properties, RPE and La in indoor climbers

## Additional Information and Declarations

### Data Availability

The following information was supplied regarding data availability: The raw data are available in the Supplemental File.
